# Emerging National Trends in Normothermic Regional Perfusion for Simultaneous Pancreas–Kidney Transplantation

**DOI:** 10.1111/ctr.70389

**Published:** 2025-11-19

**Authors:** Raphaël M. J. Fischer, Nicolas Muñoz, Olivia Ong, Peter L. Abt, Angelika C. Gruessner, Ronald F. Parsons

**Affiliations:** ^1^ Department of Surgery, Erasmus Medical Center Erasmus University Rotterdam Rotterdam the Netherlands; ^2^ Department of Surgery, Perelman School of Medicine University of Pennsylvania Philadelphia Pennsylvania USA; ^3^ Department of Nephrology SUNY Downstate Medical Center Brooklyn New York USA

**Keywords:** donation after circulatory death, kidney transplantation, normothermic regional perfusion, pancreas transplantation, perfusion, simultaneous pancreas–kidney transplantation

## Abstract

**Background:**

Normothermic regional perfusion (NRP) is rapidly gaining adoption for donation after cardiac death (DCD) organ recovery in the United States. However, little is known about trends in NRP procured grafts for simultaneous pancreas–kidney transplantation (SPK).

**Design:**

SPK recipients between January 2021 and June 2025 were identified using the United Network for Organ Sharing (UNOS)/Organ Procurement and Transplantation Network (OPTN) national data.

**Patients:**

DCD‐SPK donors and recipients were included and grouped by recovery method.

**Measurements:**

Donor and recipient demographic data were described. Primary outcomes were pancreas and kidney graft survival at 1 year, evaluated with Kaplan–Meier survival curves. Kidney outcomes included delayed graft function and creatinine levels.

**Results:**

A total of 137 DCD SPKs were included, with NRP and super‐rapid recovery (SRR) performed in 33 (24%) and 104 (76%) of donors, respectively. Donors in the NRP group were older (28 [22–34] vs. 22 [18–29], *p* < 0.05) and had a longer withdrawal‐to‐death time (22 [18–24] vs. 18 [15–22], *p* < 0.05). Recipients in the NRP group were younger (38 [35–46] vs. 48 [39‐55], *p* < 0.05), more frequently transplanted for Type 1 diabetes, and had worse functional status at the time of transplant. NRP was associated with lower rates of delayed kidney graft function (6% vs. 33%, *p* < 0.05) and a trend toward lower 6‐month creatinine (1.1 vs. 1.3 mg/dL, *p* = 0.054), with similar 1‐year values. One‐year pancreas and kidney graft survival following NRP were 91% and 100%, respectively.

**Conclusions:**

Since the introduction of NRP, 24% of the DCD‐SPK grafts were procured with NRP. Comparable 1‐year kidney and pancreas graft survival between SRR and NRP with lower rates of kidney dysfunction following NRP.

**Abbreviations**
A‐NRP: abdominal normothermic regional perfusion
BMI: body mass index
CIT: cold ischemia time
DCD: donation after cardiac death
DGF: delayed graft function
fWIT: functional warm ischemia time
KDPI: kidney donor profile index
NRP: normothermic regional perfusion
OPTN: Organ Procurement and Transplantation Network
PAK: pancreas after kidney transplantation
PNF: primary non‐function
SPK: simultaneous pancreas–kidney transplantation
SRR: super‐rapid recovery
TA‐NRP: thoracoabdominal normothermic regional perfusion
UNOS: United Network for Organ Sharing

## 1 Introduction

For patients with chronic kidney disease and insulin‐dependent diabetes, the best option for curative treatment is either simultaneous pancreas–kidney transplantation (SPK) or pancreas after kidney transplantation (PAK). SPK transplantation, the most commonly performed type of pancreas transplantation, has demonstrated excellent kidney allograft survival and insulin independence [1, 2]. In recent years, the use of donation after cardiac death (DCD) donors in the United States as a means to expand the organ pool has significantly increased [3]. Accordingly, an increase from 5894 DCD donors in 2023 to 7284 DCD donors in 2024 has been observed [4]. However, as DCD donation includes obligate periods of mal‐ and non‐perfusion, careful attention must be given to the impact of increased ischemic times on pancreas quality and subsequent transplant outcomes. As recently reported, increasing functional warm ischemia time (fWIT) and asystolic time have been shown to be significant predictors for pancreas graft loss in SPK recipients [5]. To address the risk associated with organ ischemia time, normothermic regional perfusion (NRP) has been introduced into the United States practice for DCD procurement. In NRP, perfusion of only the abdominal organs or abdominal and thoracic organs is performed in situ with oxygenated blood at a physiological temperature. NRP re‐establishes end‐organ oxygenation and reduces time pressure for dissection of the organs [6, 7]. Before NRP, DCD organs were retrieved in a super‐rapid recovery (SRR) technique, where the organ was rapidly recovered under ischemic conditions and stored until transplantation. Although SRR is still performed, the use of NRP as a means to procure organs is growing, with a more than two‐fold increase from 2022 to 2023 [8].

Currently, the only published US national registry study describing NRP in pancreas transplantation included just three pancreata [9]. The following analysis of the United Network for Organ Sharing (UNOS)/Organ Procurement and Transplantation Network (OPTN) data will therefore provide an updated assessment of NRP and SPK outcomes in the United States.

## 2 Methods

### 2.1 Study Design and Cohort

The UNOS/OPTN database was used to identify donation after circulatory death (DCD) donors, and matched recipients listed for SPK transplantation between January 1, 2021, and June 30, 2025. Donors were grouped by recovery type (SRR or NRP). The determination of whether a DCD donor underwent SRR or NRP was established using the bimodal distribution of death to cross‐clamp time with NRP defined by death to cross‐clamp ≥40 min, as previously described by Bakhtiyar et al. [10]. To classify the type of NRP, either thoracoabdominal (thoracoabdominal normothermic regional perfusion [TA‐NRP]) or abdominal (abdominal normothermic regional perfusion [A‐NRP]), the simultaneous procurement of a heart for transplantation was determined. Donor characteristics included age, sex, body mass index (BMI), cause of death, hemoglobin A1c values (HBA1C), kidney donor profile index (KDPI), death to cross‐clamp time, withdrawal‐to‐death time, pancreas preservation time, kidney cold ischemia time (CIT), ex‐situ perfusion of the kidney, and distance from donor hospital to recipient hospital. Donors were excluded if the recovery type could not be determined because of missing data. The agonal phase was defined in the UNOS/OPTN data as the time between a sustained systolic blood pressure <80 mmHg or oxygen saturation <80% and death. For recipients’ characteristics, age, sex, BMI, transplant indication, average insulin use, time on waiting list, necessity of pre‐transplant dialysis, days on dialysis, creatinine level, and functional status at time of transplant according to the Karnofsky Performance Scale were included. Pancreas outcomes were assessed by pancreas graft survival and posttransplant occurrence of abscess, anastomotic leakage, and pancreatitis. Pancreas graft failure was defined as transplant pancreatectomy, re‐registration for a pancreas or islet transplant, insulin requirement of 0.5 units/kg/day or more for 90 consecutive days, or recipient death [11]. Kidney outcomes were assessed by the occurrence of kidney primary non‐function (PNF), delayed graft function (DGF), and graft survival. Furthermore, creatinine levels at 6 months were described. DGF is defined as the requirement for dialysis during the first postoperative week, and PNF is defined as graft loss within 90 days of transplantation.

### 2.2 Statistical Analysis

Stata version 18.0 was used for analysis. Continuous data were presented as median with 25th and 75th percentile, and count and percentage for categorical data. A *p* value of 0.05 was set for significance. Primary outcomes of pancreas and kidney graft survival were described at 1 year using Kaplan–Meier survival curves.

## 3 Results

During the study period, 163 DCD SPK transplantations were performed. As a result of missing data or a death to cross‐clamp time falling in the 30‐ to 40‐min interval, 26 donors were not used for analysis as recovery type could not be determined. A total of 137 donors were included for analysis. Among the DCD donors, NRP was performed in 33/137 (24%) and SRR in 104/137 (76%). The withdrawal‐to‐death time was significantly longer for donors in the NRP group (22 [18–24] vs. 18 [15–22], *p* = 0.012) while the agonal phase was similar (15 [12–20] vs. 15 [11–19], *p* = 0.37). When identifying the type of NRP, 25/33 (76%) of donors were procured with TA‐NRP and 8/33 (24%) with A‐NRP. Donors in the NRP group were older (28 [22–34] vs. 22 [18–29], *p* < 0.001) while other characteristics were comparable between the two groups (Table 1). The liver was recovered in 97% of the NRP cases as opposed to 89% of SRR cases. Kidneys from the SRR group were subjected to longer cold ischemic times than the NRP‐recovered kidney (12 [9–15] vs. 9.3 [6.7 vs. 12], *p* = 0.017). Compared to recipients of SRR grafts, recipients in the NRP group were younger (38 [35–46] vs. 48 [39–55], *p *= 0.001) and were more often transplanted for Type 1 diabetes. Additionally, the NRP group had a higher percentage of disabled or very disabled recipients at time of transplant. The requirement for pre‐transplant dialysis was comparable between the two groups.

**FIGURE 1:**
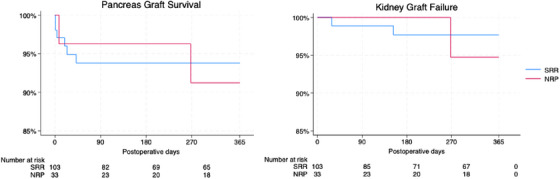
Kaplan–Meier survival curves for pancreas and kidney graft survival. Pancreas and kidney graft survival after simultaneous pancreas–kidney transplantation following donation after super‐rapid recovery and normothermic regional perfusion.

In terms of postoperative complications, comparable outcomes were observed between the two groups (Table 1). However, the SRR group had an increased incidence of kidney DGF (32% vs. 6%, *p* = 0.007). Use of ex‐situ machine perfusion was 35% and 36% in SRR‐kidneys and NRP‐kidneys, respectively. PNF occurred in one SRR‐procured kidney and in none of the NRP kidneys. Creatinine levels at 6‐month posttransplantation had a trend toward lower values in recipients from NRP grafts (1.1 mg/dL [1.1–1.5] vs. 1.3 mg/dL [1.1–1.5], *p* = 0.054), with similar 1‐year creatinine values between both groups.

**TABLE 1:** Donor and recipient characteristics for SRR and NRP groups.

		SRR	NRP	*p* value
Donor		*N* = 104	*N* = 33	
Age		22 (18–29)	28 (22–34)	**<0.001**
BMI		23 (21–25)	24 (21–26)	0.35
Sex				
	Male	76 (73%)	14 (78%)	0.74
	Female	21 (26%)	4 (22%)	
Cause of death				
	Anoxia	34 (42%)	16 (49%)	**0.006**
	Head trauma	40 (49%)	10 (30%)	
	CVA	4 (4%)	7 (21%)	
	Other	5 (5%)	0 (0%)	
HBA1C		5.3 (5–5.5)	5.3 (5–5.5)	0.96
KDPI		0.21 (0.11–0.029)	0.25 (0.19–0.31)	0.12
Withdrawal‐to‐death time (min)		18 (15–22)	22 (18–24)	**0.012**
Agonal phase (min)		15 (12–20)	15 (11–19)	0.37
Death to cross‐clamp time (min)		6 (5–9)	94 (74–114)	**<0.001**
Liver recovered		92 (89%)	32 (97.0%)	0.15
Pancreas preservation time (h)		11 (8.9–14)	10 (7.6–12)	0.065
Kidney cold ischemia time (h)		12 (9–15)	9.3 (6.7–12)	**0.017**
Kidney ex‐vivo machine perfused				
	Yes	36 (35%)	12 (36%)	0.43
	Unknown	6 (6%)	4 (12%)	
Donor to recipient distance (nautical miles)		141 (28–233)	75 (22–220)	0.89
Recipient				
Age		48 (39–55)	38 (35–46)	**0.001**
BMI		27 (24–29)	25 (22–29)	0.73
Sex				0.063
	Male	72 (69%)	17 (52%)	
	Female	32 (31%)	16 (48%)	
Pancreas diagnosis				**0.002**
	Type 1 diabetes	56 (54%)	23 (70%)	
	Type 2 diabetes	47 (45%)	7 (21%)	
	Unknown	1 (1%)	3 (9%)	
Insulin (units/kg/day)		0.48 (0.32–1.1)	0.42 (0.29–0.7)	0.22
Time on waiting list		161 (49–496)	141 (36–545)	0.74
Pretransplant dialysis				0.24
	Yes	83 (79.8%)	28 (84.8%)	
	Unknown	2 (1.9%)	2 (6.1%)	
Days on dialysis		734 (426–1120)	579 (433–963)	0.55
Creatinine		7.5 (5.4–9.3)	6.5 (5.4–7.8)	0.23
Functional status				**0.032**
	Very disabled	7 (7%)	3 (9%)	
	Disabled	2 (2%)	4 (12%)	
	Normal/symptomatic	83 (80%)	18 (55%)	
	Other/ Unknown	7 (7%)	6 (18%)	
Length of stay		9 (6–13)	8 (6–12)	0.34
Abscess				1.00
	Yes	3 (3%)	1 (3%)	
	Unknown	9 (9%)	6 (18%)	
Anastomotic leak				0.45
	Yes	11 (11%)	1 (3%)	
	Unknown	10 (10%)	6 (18%)	
Pancreatitis				0.81
	Yes	7 (7%)	1 (3%)	
	Unknown	9 (9%)	6 (18%)	
Kidney delayed graft function				0.007
	Yes	33 (32%)	2 (6%)	
	Unknown	7 (7%)	6 (18%)	
Primary non‐function				0.80
	Yes	1(1%)	0 (0%)	
	Unknown	11 (11%)	9 (27%)	
Creatinine at 6 months		1.3 (1.1–1.5)	1.1 (1.0–1.3)	0.054
Creatinine at 1 year		1.3 (1.1–1.5)	1.1 (1.0–1.3)	0.22

*Note:* Data are presented as median (p25, p75) and *n* (%).

When analyzing pancreas graft failure, two graft failures were observed in the NRP cohort during the first‐year posttransplantation. Pancreas graft survival analysis showed comparable outcomes between SRR and NRP at 180 days (94% vs. 96%, *p* = 0.60) and 365 days (94% vs. 91%, *p* = 0.87). One kidney graft failure was observed in the NRP group during the first year‐months posttransplantation. Kidney graft survival analysis showed comparable outcomes between SRR and NRP at 180 days (98% vs. 100%, *p* = 0.46) and 365 days (98% vs 95%, *p* = 0.60) (Figure 1).

## 4 Discussion

Evidence suggests that transplant centers have neglected pancreas transplantation in recent decades and strategies for a resurgence include reassertion of the importance of pancreas procurement, especially during DCD donation [12]. The introduction of NRP has rapidly changed the field of transplantation following DCD donation. In this analysis of the UNOS/OPTN database investigating the role of NRP in SPK transplantation, we identified 137 DCD‐procured pancreata with 33 (24%) donors undergoing NRP and 104 donors (67%) undergoing SRR. In 97% of the NRP cases, the liver was procured compared to 89% in the SRR cases. Overall, similar kidney and pancreas graft survival were observed at 180 and 365 days, with NRP‐procured kidneys showing lower rates of DGF and a trend toward lower 6‐month creatinine levels.

Literature on the early experience of SPK transplantation from NRP DCD donors is scant and still emerging. A case series by Gómez‐Dueñas et al. [13] describing four recipients undergoing SPK following NRP demonstrated 1‐year pancreas and kidney graft survival of 75% and 100%, respectively. An analysis of the UK Transplant Registry reported that 11% of the DCD‐SPK were procured with NRP, resulting in higher 1‐year pancreas graft survival in the NRP group, though not statistically significant [14]. In the United States, an early analysis of the UNOS/OPTN data by Bekki et al. [9] described the use of NRP in three donors, accounting for 0.7% of the 44 DCD pancreas transplantations at the time. Our analysis indicates that 24% of DCD pancreata were procured through NRP. The increased proportion likely represents the growth of NRP procurement generally within the United States [8]. We have seen substantial adoption of NRP with approximately 84%—up from 51% in 2024—of DCD donors in our organ procurement organization recovered through NRP as of May 26, 2025 [15].

DGF of the kidney has been shown to be associated with an increased risk of pancreas dysfunction and failure [16, 17]. Furthermore, kidney DGF has recently been associated with postoperative complications and death‐censored graft failure of the pancreas in SPK recipients [18]. Given these concerns, NRP could be a key strategy to limit kidney DGF and improve pancreas graft survival. In our study, we report lower rates of kidney DGF in NRP procured grafts (6% vs. 32%, *p* = 0.007). Interestingly, the DGF rate we found in the NRP group is lower than SPKs from brain‐dead donors [19, 20]. Additionally, kidney DGF has been associated with elevated 1‐year creatinine levels [17]. In our study, there was a trend toward higher 6‐month creatinine levels in the SRR group, potentially linked to the greater rate of DGF in this group.

One limitation in this study is the lack of a specific variable for NRP in the UNOS/OPTN database. The designation of NRP procured organs according to the death to cross‐clamp time, while not without flaws, has been accepted previously as a method to identify NRP donors [10]. The absence of a variable for NRP is reflected in the exclusion of 27 recipients for whom determining the recovery type of the donor was not possible. Although 33 donors may accurately reflect the smaller number of pancreata procured with NRP compared to SRR, the small sample size influences the statistical analysis. Furthermore, data on NRP procedure‐specific details, such as start or end time of NRP, are lacking. These variables are crucial as they are necessary to calculate the donor fWIT, which plays an important role in DCD donation and the total perfusion time. As seen in rapid‐recovered pancreata, longer donor fWIT is significantly associated with pancreas graft loss. Whether the negative impact of longer donor fWIT is alleviated by the use of NRP is still to be determined. As such, proper identification of donors undergoing NRP, as well as including data on the recovery procedure, is necessary. As of October 1, 2025, the UNOS/OPTN has updated the Deceased Donor Registration form to include crucial data in DCD donation and NRP‐procurement. Likewise, identification of pancreas graft failure is center reported, and its diagnosis may not be made consistently. Pancreas graft failure may be underreported and could influence the results in this and other registry studies. As the use of NRP continues to expand, with both more SPK from NRP donors and additional follow‐up time, future studies should have even greater power to detect clinically significant differences in outcomes.

In conclusion, when comparing DCD donors by recovery type, there are increasing proportions of DCD SPK performed with NRP as compared to SRR, and outcomes between SRR and NRP were overall equivalent. Although pancreata and kidneys showed similar graft survival during the initial months posttransplant, our results suggest that there is less graft dysfunction among NRP‐procured kidneys, as there was less DGF and a trend toward lower 6‐month creatinine levels compared to SRR. The possibility of improved pancreas graft survival following NRP versus SRR after less kidney DGF warrants ongoing investigation. In this analysis, pancreas graft survival was similar at 1 year. Overall, NRP results in lower posttransplant kidney dysfunction and excellent pancreas function. However, we strongly advocate for the introduction of an NRP‐specific variable in the UNOS/OPTN database to ensure that future analyses are scientifically sound and can be used with confidence to optimize clinical practice.

## Author Contributions

**Raphaël M. J. Fischer**: Study conception, study design, data analysis, writing of manuscript, submission of manuscript. **Nicolas Muñoz**: study conception, study design, data analysis, writing of manuscript. **Olivia Ong**: data analysis. **Peter L. Abt**: study conception, study design, review of manuscript. **Angelika C. Gruessner**: study design, review of manuscript. **Ronald F. Parsons**: study conception, study design, review of manuscript, writing of manuscript.

## Conflicts of Interest

RFP receives institutional research funding support from Natera, Transplant Genomics, ITB‐Med, Regeneron, Hansa Biopharma, is a member at large for the AST Living Donor Circle of Excellence, is chair of the American Society of Transplant Surgeons Cellular Transplantation Advisory Committee and is a member of the Pennsylvania Gift of Life Medical Advisory & Policy Board.

## Data Availability

The data that support the findings of this study are available on request from the corresponding author. The data are not publicly available due to privacy or ethical restrictions.
